# Knowledge, attitude, and practice in diagnostic dental radiology: an Egyptian survey with a global perspective

**DOI:** 10.1038/s41405-026-00415-2

**Published:** 2026-04-11

**Authors:** Mohamed F. Rashed, Lamia Khairy Gadallah, Mohamed Abdelfattah Galal, Khaled Helmi El-Wakeel, Hend S. ElSayed

**Affiliations:** 1 Orthodontics and Pediatric Dentistry Department, Oral and Dental Research Institute, National Research Centre, Giza, Egypt; 2 Surgery & Oral Medicine Department, Oral and Dental Research Institute, National Research Centre, Giza, Egypt; 3 Biological Anthropology Department, Medical Research and Clinical Studies Institute, National Research Centre, Giza, Egypt

**Keywords:** Digital radiography in dentistry, Occupational health

## Abstract

**Objectives:**

Many surveys report less than optimum knowledge, attitude, and practice (KAP) of dental imaging for patient and operator safety. This study aimed to assess the KAP for diagnostic imaging among dentists in light of global practices and recent recommendations.

**Methods:**

A cross-sectional survey was conducted using a structured questionnaire that was electronically distributed through dental social media platforms between March 1st and June 30th, 2022. Forty-seven questions evaluating demographics, knowledge, practice, and attitude were included. The survey targeted Egyptian dentists practicing in Egypt.

**Results:**

Two hundred and twenty-eight dentists of different specialties responded. Fifty-nine percent of the dentists were not familiar with the ALARA principle. Fifty-three percent never use lead aprons. Dentists (16.6%) who practiced in facilities with cone-beam computerized tomography (CBCTs) showed significantly (*p* = 0.000) higher knowledge scores (7.89 ± 2.63) than those who didn’t have CBCTs (6.16 ± 2.67). There was no statistical difference in training received (*p* = 0.345) between the different specialties. Most dentists (79.4%) were interested in increasing their knowledge of radiation safety.

**Conclusions:**

Within the limitations of this study, there is a need to educate dentists on radiation hazards, child-sized imaging protocols, and how to implement the ALARA principle. Training materials freely available on reliable websites should be disseminated. Regulations should include auditing referrals and periodic calibration of X-ray units.

## Introduction

There is an international consensus regarding three fundamental concepts for radiation protection. These are the justification of the procedure, optimization of the technique and dose limitation [1]. The World Health Organization (WHO) [2] and the American Dental association (ADA) [3] recommend that clinicians exert every effort to minimize the radiation exposure even for low-level dental x-ray imaging [4, 5]. International bodies and initiatives have provided information and training materials for patients, guardians and health care providers, to help reduce unnecessary radiation exposure [6, 7] especially to children [8].

Despite the global movement to establish a radiation safety culture in healthcare facilities and among providers, most studies continue to report suboptimal knowledge and practices of radiation protection. Previous surveys in different regions of the world have shown little awareness of the recommendations of leading international organizations [9]. Some studies show inconsistent use of protective wear [10], inappropriate use of radiographic techniques [11], little practice auditing [12], as well as lack of machine maintenance and calibration [13].

In dentistry, X-rays are the major source of ionizing diagnostic radiation [5]. Although it is responsible for low dose exposures, there are approximately 1.5 billion dental radiographic examinations, annually. These account for 10-40% of global health care exposures.

The increase in number of exposures over the last two decades, as well as the use of the Cone-Beam Computerized Tomography (CBCT) which produces 10–20 times the radiation dose of conventional modalities [4], warrant additional safety measures.

The biological effects of ionizing radiation are rare in dental radiography. However, children are more sensitive to radiation [14, 15]. They are also more likely to need dental X-rays since dental trauma, caries and orthodontic treatment are more prevalent in younger patients. This emphasizes the importance of raising the awareness of radiation safety and hazards among different dental specialties, particularly those dealing with children and adolescents [1].

Recently several leading dental bodies have updated the recommendations for safe diagnostic imaging [16]. These updates signify changes to some aspects of imaging in the dental practice. The aim of this study was to evaluate the knowledge, attitude and practice (KAP) of radiation safety and hazards among dental specialties and highlight global practices and recent recommendations in dental radiography.

## Methods

This study is a cross-sectional survey that was designed as a part of a project to build a radiation safety culture at the National Research Centre (NRC), Egypt. The study received the ethical approval (ID: CT01032023) from the Medical Research Ethics Committee, NRC, before the start of the study. The survey was distributed electronically, between March 1st and June 30th, 2022, as a Google Form. The questionnaire was shared to eight dental social media platforms and WhatsApp messenger groups, in Egypt.

### Questionnaire development

The project’s research team were assigned a national or international guideline to evaluate and identify the aspects of basic knowledge and practice of dental radiation hazards and safety and develop questions regarding this data. The examined guidelines included those of the International Commission on Radiological Protection, American Academy of Pediatric Dentistry (AAPD), the European Union (EU) and the WHO, as well as the International Atomic Energy Agency (IAEA) radiation safety curriculum and recommendations. Seventy-five questions were presented to a team of 10 dental specialists. The dentists were invited to select the questions that represent essential knowledge and practice aspects. Questions that were agreed upon by 80% of the dentists were considered for round two of the Delphi. In the second-round, dentists were requested to select what they deemed the most relevant questions to knowledge and practice.

The questionnaire consisted of 47 questions: demographic data (9), knowledge (23), practice (12), attitude (2) and one question of self-assessed knowledge (1). Two verification questions were included to test consistency of the collected information. Verification was tested for the ALARA concept and the dentist’s knowledge. We asked “Do you know what ALARA stands for? (Yes/No)”. This was verified with a multiple-choice question “The ALARA principle means using radiation As Low As Reasonably? (Affordable, Achievable, Acceptable)”. Knowledge was tested by asking the participants to self-assess their level of knowledge and this was verified by correlating the answers with the actual total knowledge score out of 23. The knowledge questions covered radiation physics, hazards and protection.

The completed questionnaire was piloted by thirteen volunteers of different dental specialties. They answered the questionnaire, and the concept behind each question was explained to them. They were asked to score if the questions represented the targeted concepts on a scale of 1–10. The concordance correlation coefficient for these questions was 0.850 with a confidence interval 0.626, 0.945. Some dentists suggested rephrasing some questions for clarity. To assess attitude the participants’ previous training in radiation safety and interest in future training were evaluated. These steps established the content and face validity of the survey.

Previous surveys [17–19] used multiple-choice questions with a single correct answer where 1 mark was awarded for each question. In this survey, questions had multiple correct answers, and all possible options had to be selected for the question to be considered correct.

### Sample size calculation

The sample size was calculated based on the following formula: *n* = (4Z^2^ pq)/d^2^. The total population of dentists, in Egypt, at the time of the survey was 85,000. The sample size was estimated at 246 dentists, [*n* = sample size, *Z* = 1.96, *P* = 0.20, *q* = 0.80 (1-p), *d* = 5% margin of error]. The post-hoc margin of error calculated for this study was 5.2% for 228 participants with a 95% confidence interval and 18% of the population showing radiation safety knowledge.

### Statistical analysis

The data was coded then analyzed using PASW Statistics 18. Descriptive data were expressed as frequency, percentages, means and standard deviations. Spearman’s correlation coefficient was calculated for the knowledge score and self-assessed knowledge. Kruskal-Wallis test compared the knowledge score between different dental specialties. Pearson Chi-Square test was used to compare attitudes between dentists with different affiliations, as well as attitudes between different specialties. All tests were two-tailed.

### Ethical statement

The study received the ethical approval (ID: CT01032023) from the Medical Research Ethics Committee, National Research Centre. The study was performed in accordance with the ethical standards as laid down in the 1964 Declaration of Helsinki and its later amendments.

## Results

Two hundred and forty-six dentists responded to the questionnaire. The data for ten non- Egyptian participants was excluded. Multiple submissions of the survey by the same participant were identified from their emails and were excluded. Most respondents represented the Cairo and Giza governorates.

### Demographics

The distribution of the dentists’ affiliations, specialties, years of experience and gender are shown in Table 1.

**Table 1:** Demographic data for participating dentists.

	Participating dentists *n* = 228		
	Categories	Frequency	Percent
Gender	Male	59	25.9
	Female	169	74.1
Specialties	Undefined	9	3.9
	Oral Radiologist	18	7.9
	Pedodontist	41	18.0
	GP	47	20.6
	Others	113	49.6
Affiliation	Research Organization	74	32.5
	University	92	40.4
	MOH	21	9.2
	Educational Hospital	20	8.8
	Private practice	21	9.2
Years of experience	1–4 years	32	14.0
	5–9 years	26	11.4
	10–14 years	53	23.2
	15–19 years	59	25.9
	≥20 years	58	25.4

*MOH* Ministry of Health, *GP* General dental practitioner.

### Actual knowledge scores

**Fig. 1:**
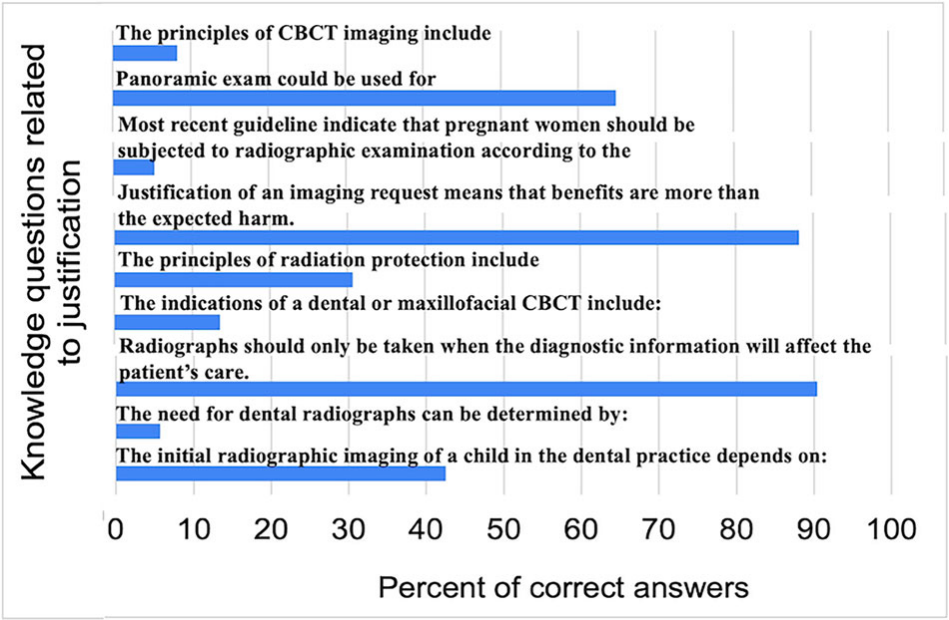
Percent of correct answers for knowledge questions related to justification of imaging.

**Fig. 2:**
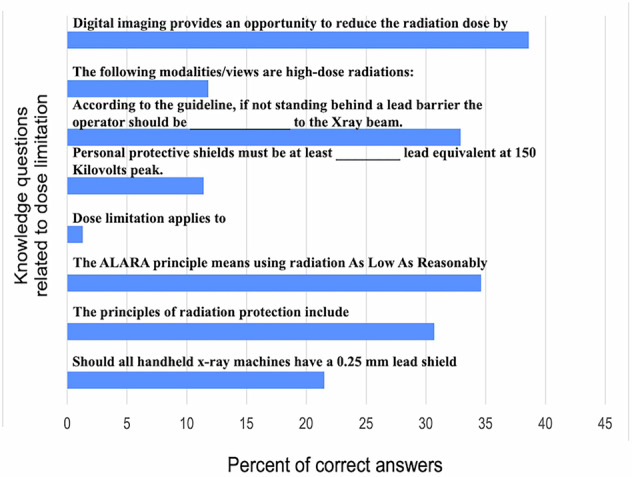
Percent of correct answers for knowledge questions related to dose limitation.

**Fig. 3:**
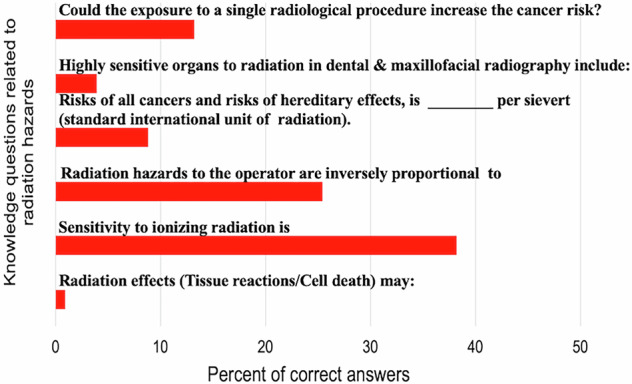
Percent of correct answers for knowledge questions related to radiation hazards.

Most knowledge questions were answered correctly by less than 50% of the dentists. The mean score for correct answers was 6.48 ± 2.71 out of 23. Figures 1–3 show the results for questions related to justification of imaging, related to dose limitation, and related to radiation hazards, respectively. The minimum score was 1 and the maximum was 19. The lowest scores were recorded for questions related to the hazards of radiation as shown in Fig. 3.

**Fig. 4:**
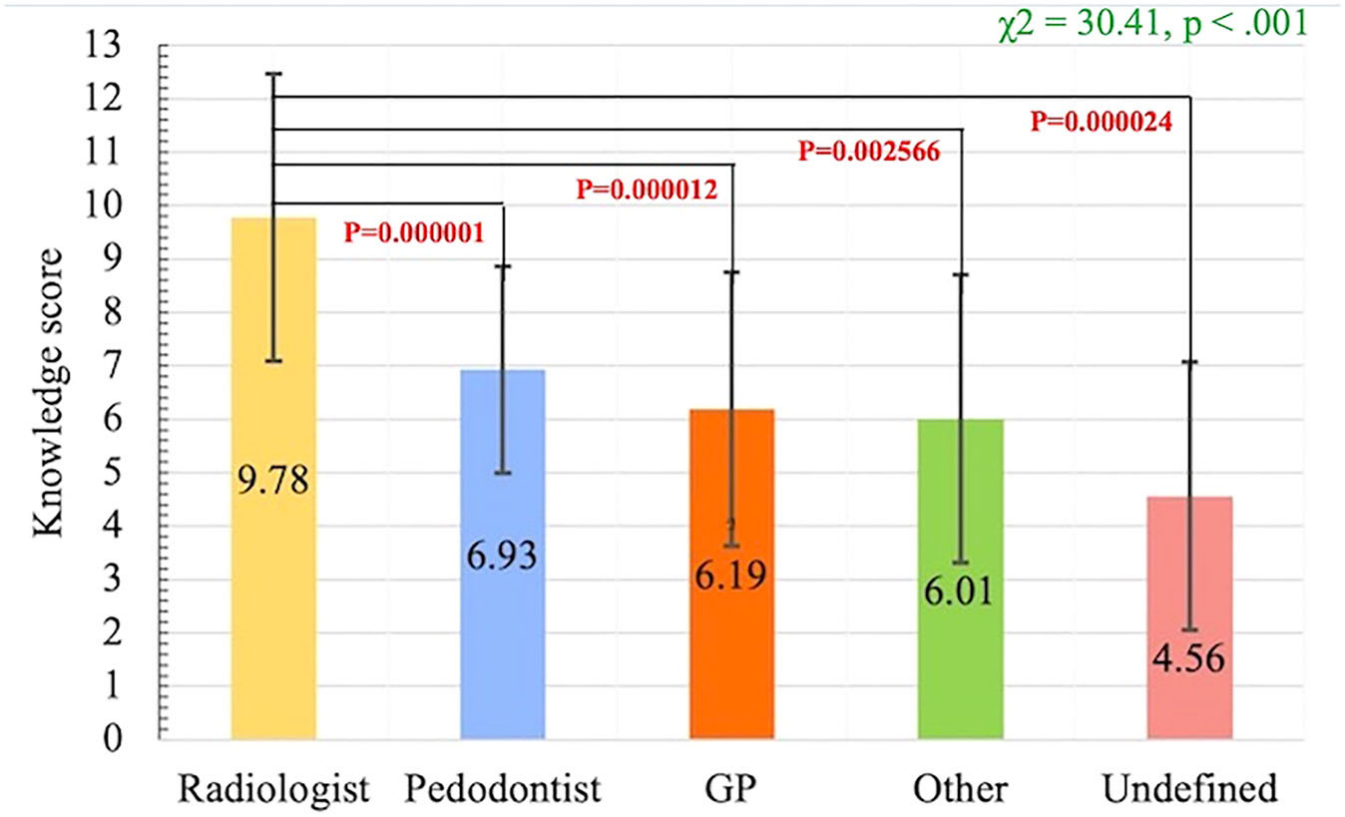
Comparison between knowledge scores in different dental specialties.

There was no significant difference (*p* = 0.610) between the knowledge scores for male (6.64 ± 2.77) and female (6.38 ± 2.73) dentists. Non-significant differences were shown between the knowledge scores for different years of experience (mean; 6.45, *p* = 0.957) and different affiliations (mean; 6.45, *p* = 0.138). However, there was a significant difference (*p* = 0.000) in the knowledge scores between different specialties (Fig. 4). Dentists who have CBCTs (16.6%) in their facilities showed significantly higher knowledge scores (7.89 ± 2.63) than those who didn’t have CBCTs (6.16 ± 2.67) (*p* = 0.000).

The results show insignificant correlations between years of experience and actual knowledge scores or self-assessed knowledge as shown in Table 2. A significant correlation was shown for the actual knowledge score and the self-assessed knowledge in Table 2. However, the actual scores did not reflect the level of self-assessed knowledge as shown in Table 3.

**Table 2:** The Spearman’s correlation coefficient between the actual knowledge score of the dentists and the self-assessed knowledge, and years of experience.

		Actual knowledge Score *n* = 228	Years of experience *n* = 228	Self-assessed knowledge *n* = 228
Actual knowledge Score		1.000	−0.027	0.302^**^
	*p*-value		0.680	0.000
Years of experience		−0.027	1.000	0.083
	*p*-value	0.680		0.210
Self-assessed knowledge		0.302^**^	0.083	1.000
	*p*-value	0.000	0.210	

**Correlation is significant at *p* = 0.01.

**Table 3:** Descriptive statistics for the self-assessed knowledge and the actual knowledge score for the dentists.

	Self-assessed knowledge reported by the dentists Frequency (%)	Actual knowledge score of the same dentists (Number of correct answers for the knowledge section out of 23) Mean ± SD
I know very little	19 (8.4)	4.35 ± 2.21
I know the basics	162 (71.0)	6.32 ± 2.58
Very knowledgeable	39 (17.1)	7.77 ± 2.47
Expert	8 (3.5)	8.0 ± 4.54

### Radiation facility equipment, requirements, audits, and regulations

Twenty-three facilities had no dental X-ray unit, while three had an X-ray unit, panorama, CBCT, and a handheld X-ray machine. One hundred and twenty-three facilities had at least one X-ray unit. Twenty-five facilities had a Dental X-ray unit, and a panorama, while twenty-six facilities had Dental X-ray unit, panorama, and CBCT. Seven facilities had a handheld X-ray machine.

Sixteen percent of dentists were unaware if their facilities had wall and door radiation shields, while 53.5% of their facilities were shielded and 30.0% were not. Thirty percent of facilities had signs denoting the radiation zones, while 52.0% did not and 17.5% of dentists did not know if a sign was available.

Sixty-four percent of the dentists did not know if X-ray units in their facilities were being calibrated. Some machines were calibrated every 6 months (7%), annually (10.1%), and every 3 years (3.1%), while some had not been calibrated in the previous 5 years (15.8%).

Radiography referral requests were audited in 33.8% of facilities. While 32.9% of the dentists were not aware if referrals were audited in their facilities.

### Occupational safety, personal protective equipment and monitoring

Dentists (42.5%) didn’t know where to stand if a barrier was not present. Fifty-three percent, 81%, and 79.4% of dentists never wear lead aprons, goggles or thyroid collars, respectively. There was a significant difference between the use of lead apron (*p* = 0.002) among radiologists (88.9%), pedodontists (29.3%), other Specialists (47.8%), and general dental practitioners (GP). Similarly, the use of goggles was significantly different (*p* = 0.003) among radiologists (27.8%), pedodontists (12.2%), other specialties (19.5%), and GP (10.6%). The use of thyroid collar among radiologists (66.7%), pedodontists (16.4%), other Specialties (13.3%), and GP (21.3%) was statistically different (*p* = 0.000). Sixty-nine percent of the dentists did not monitor possible hazards of radiation. Those who were monitored used film badges (17.5%) and Periodical examination (10.5%).

Twenty-one percent of the dentist answered correctly regarding the lead shield thickness (0.25 mm) required for handheld machines, while 75% didn’t know. Two out of the 7 dentists who have handheld X-ray machines answered this question correctly.

### Dentist’s knowledge of radiation hazards

Thirty-nine percent were aware that sensitivity to ionizing radiation was highest in prenatal life and children were more sensitive than adults. Only two dentists knew the difference between the deterministic and stochastic effects of radiation. Seventy-five answered incorrectly to the factors that are inversely proportional to radiation hazards. Dentists (81%) did not know the risk of cancer and hereditary effects. One dentist identified all the sensitive organs in the head and neck region. Twenty-four percent thought that a single dose of radiation is a cancer risk only at elevated exposure doses. Fourteen percent answered incorrectly to what are the principles of radiation protection.

### Dentist’s radiation safety practice towards the patients

A third of the dentists knew what ALARA stands for. Most Dentists (90%) agreed that radiographs should only be taken when the information will affect the patient’s care. Sixty-four percent believed dose limits were for patients as well as health professionals and the public.

Some dentists (3–8%) requested radiographic imaging: before the clinical examination, as routine imaging at the first visit and at checkups every 6 months. Only 6% considered the patients’ medical and dental history, clinical examination and exposure to other sources of radiation when requesting a radiograph.

Dentists were not clear on which procedures are low-dose exposures. Some believed that only full skull CBCT produced high-dose radiation. On the other hand, few dentists considered periapical, bitewing, OPG and lateral cephalometry as high-dose images. Sixteen percent of dentists knew the indications of the CBCT. While seventeen out of the 228 dentists considered the CBCT for routine examination.

Forty-one percent of the dentists were unaware of the type of film or sensor being used in their facilities, while digital sensors (37.8%) and radiographic films (38.7%) were similarly used. Dentists used D, E and F speed films in a ratio of 4.7:2.7:1, respectively. The dentists report that during radiation exposure, the film is usually held by the patient followed by the operator, parent and least frequently the film holder.

### Dentist’s radiation safety practice towards children and pregnant women

Twenty four percent of the dentists thought the initial radiograph in children depended on age. A similar number answered, “I don’t know”. A few dentists (17) considered the OPG for imaging children in the primary dentition. Most dentists (66%) responded “I don’t know” to the protocol for imaging pregnant women. Pedodontists were the least to use film holders.

### Attitude towards continuing education in radiation protection

Dentists who received training in radiation safety and hazards in universities, research organizations, private practices, MOH and educational hospitals were 39.5%, 34.2%, 18.4%, 5.3%, 2.6%, respectively. There was no statistical difference for the training received (*p* = 0.137) by affiliation, or by specialty (*p* = 0.345). Most dentists received training as undergraduates, and only radiologists had advanced training in radiation safety.

The attitudes towards continuing education of radiation safety and hazards by affiliation and specialty were reported in Tables 4 and 5, respectively. There was no difference in the willingness of dentists to receive continuing education by affiliations (*p* = 0.423) or by specialty (*p* = 0.150). Nine out of 228 dentists were not willing to attend CE.

**Table 4:** Descriptive statistics of dentist’s attitudes towards continuing education of radiation hazards and protection by affiliation.

Dentists’ response to if they would agree to attend continuing education *n* = 228						
	Yes		No		Maybe	
Affiliation	Frequency & percent	Percent within the affiliation	Frequency & percent	Percent within the affiliation	Frequency & percent	Percent within the affiliation
Research Organization	58 (32.0%)	78.40%	6 (66.7%)	8.10%	10 (26.3%)	13.50%
University	75 (41.4%)	81.50%	2 (22.2%)	2.20%	15 (39.5%)	16.30%
MOH	16 (8.8%)	76.20%	0 (0.0%)	0.00%	5 (13.2%)	23.80%
Educational Hospital	14 (7.7%)	70.00%	1 (11.1%)	5.00%	5 (13.2%)	25.00%
Private Practice	18 (9.9%)	85.70%	0 (0.0%)	0.00%	3 (7.9%)	14.30%

**Table 5:** Descriptive statistics of dentist’s attitudes towards continuing education of radiation hazards and protection by specialties.

Dentists’ response to if they would agree to attend continuing education *n* = 218^a^						
	Yes		No		Maybe	
Specialty	Frequency & percent	Percent within the affiliation	Frequency & percent	Percent within the affiliation	Frequency & percent	Percent within the affiliation
Radiologists	16 (8.8%)	88.90%	0 (0.0%)	0.00%	2 (5.3%)	11.10%
Pedodontists	38 (21.0%)	92.70%	0 (0.0%)	0.00%	3 (7.9%)	7.30%
Other Specialists	81 (44.8%)	71.70%	8 (88.9%)	7.10%	24 (63.2%)	21.20%
GP	38 (21.0%)	80.90%	1 (11.1%)	2.10%	8 (21.1%)	17.00%

^a^one non-responder, 9 dentists with unidentified specialty.

## Discussion

Radiographic imaging is important for the diagnosis, and treatment planning of dental patients. Despite the low-dose exposure involved in dental radiography, cumulative effects of ionizing radiation from medical and other sources should be considered [4, 5].

The increasing use of ionizing radiation [4], the insufficient training of medical staff [18, 20], and the continuous emergence of new technologies warrants the periodical assessment and training of healthcare providers [4] In this survey, the knowledge and practice were investigated to better understand when and how dentists request and image dental patients, to better inform them and policy makers on safer practice and training programs.

On average, only twenty-eight percent of the knowledge questions were answered correctly in this survey. This differs from the results reported by Furmaniak et al. in Poland [17], Yurt et al. in Turkey [18], and Aljamal et al. in Palestine.[21] at 8.13 out of 13 questions, 8.3 ± 2.1 out of 17 and 7.5 ± 2.67 out of 16, respectively. The low scores in our survey may be attributed to the method the questions were marked. All correct options had to be selected for the question to be marked as correct. This may have underestimated the knowledge scores.

This study as well as studies carried out in Turkey [18] and Wales [22] reported that years of experience, the affiliations and gender were not associated with the knowledge score. Only the specialty of the participants was. On the other hand, one study in India showed that higher scores 9.54 ± 2.54 (out of 16 questions) were associated with academicians [19]. They attributed this to their continuing education and their frequent exposure to scientific literature. A survey in Palestine reported that 4th and 5th year students had higher knowledge scores than junior students and practicing dentists [21]. The authors suggest that the students’ recent exposure to training account for their better scores. A survey in Iraq reported higher knowledge for dental specialists compared to general practitioners [23].

Similar to the survey carried out in Uganda [24], “I don’t Know” was a frequent answer, for some questions. Previous surveys similarly reported confusion and lack of clarity of radiation safety procedures and policies [20, 25]. Furmaniak et al. suggest that “I don’t know” answers could underestimate knowledge scores since they are counted as incorrect answers [17].

The results of this study imply a lack of clear communication between dental facility management and the health care providers. Operators in up to sixty-four percent of facilities were not aware of the type of barriers in the radiation area, or if X-ray units were calibrated. Our results showed that 50% of facilities lacked warning signs which may present a hazard. However, a survey in Turkey [18] reported that 96.9% of facilities had radiation warning sign. Which may be related to regulations or education.

This study showed that 41% of the participating dentists were unaware of the type of film or sensor used in their facilities, this differs from the results of an Indian surveys [26], where only 4% did not have any knowledge about the speed of film they used.

Concerning dose limitation for operators, the common rule of distance, time and shield should be implemented if barriers are not available. The 6 feet (≈2 m) distance at an angle of 90–135° from the central ray of X-ray beam should always be kept [3]. Due to presence of barriers, the results of our survey showed that only a third of participating dentists knew this rule. Operators who are exposed to larger doses of occupation radiation need to observe shielding with lead aprons and even thyroid collar according to their workload. In this study, oro-maxillofacial radiologists reported the highest use of these shields which is in line with their higher workload. Similarly, the use of thyroid collars and the lead aprons was 66.9% and 80.2%, respectively, among Saudi dentists [27]. Panchbhai and Sonar [28] reported that 71.4% of dentists wore personal protective devices.

Occupational radiation monitoring varied widely between surveys. In Korean dentists, monitoring was related to years of experience [29] While an Indian survey [26] showed that utilization of dosimeters among specialists (16.2%) was higher compared to nonspecialists (1.7%). In this survey, few dentists reported periodic examination (5%) and the use of film badges (10%) to monitor radiation exposure. Which may be associated with less frequent imaging. The expected occupational dose determines the need for dosimeters, yet surveys didn’t correlate them.

Few dentists in this survey operated handheld X-ray machines and they did not take appropriate precautions. These machines have a higher output voltage to overcome poorer image quality, and they generate more scattered radiation. Therefore, they should only be considered when conventional facilities are unavailable and with special considerations [30].

Deterministic effects of ionizing radiation are documented, yet rare in dental radiography [31], yet the stochastic effects are still possible according to the non-linearity theory [30]. The sensitivity of different organs must be considered when assessing radiation hazards. The head and neck region houses the salivary and the thyroid glands, as well as the eyes which are among the radiation sensitive organs [32]. In the study by Talha et al. [33], the Parotid gland and oral mucosa absorbed the highest radiation during panoramic and CBCT imaging. Notably, the tissue weighted factor for both are high. Our survey and Shahab et al. [34] showed that few participants were aware of the sensitive tissues in the oro-facial region.

The ALARA principle is a cornerstone of medical imaging. It is recommended by all radiation protection organizations since 1977. The use of as low as reasonably achievable radiation doses must always be implemented, particularly in children [35]. However, more recently in 2015, the ALADA “As Low As Diagnostically Acceptable” principle was presented as a response to the introduction and routine use of CBCT in the dental practice. It is refined from the ALARA principle to reach a diagnostically acceptable and interpretable image highlighting the optimization of the image quality, as well as the reduction of the radiation dose [36–38]. In 2017, the ALADAIP “As Low As Diagnostically Acceptable being Indication-oriented and Patient-specific” concept was introduced for personalized optimization with an eye on pediatric patients [39]. However, among these concepts the ALARA principle is the most commonly mentioned in published surveys and was therefore assessed in our questionnaire to allow comparisons. In this survey, thirty-five percent of dentists knew the principal in comparison to 84.3% and 68.1% of dentists from India [40] and Saudi Arabia [27] respectively.

Digital radiography and sensors play an important role in minimizing radiation. Compared to conventional imaging, digital radiography can reduce up to 80% of the exposure with enhancement of image quality [41]. Intraoral sensors were used by 38% of Flemish GPs [42], 65.4% of dentists in Hong Kong [43], and 67% of Turkish dentists [44]. While digital radiography was used by 70% of specialists and 30% of GPs in the UK [45]. In our survey, dentists used sensors and X-ray films alike. Low-middle income countries, show lower adoption of digital technologies, which may be attributed to socio-economic levels [44].

Most dentists participating in this survey were aware that radiographs should only be taken if they will further inform the patients’ health care. However, poor practices of patient safety were observed. Three to eight percent of dentists took radiographic images: before the clinical examination, routinely at the first visit and every 6 months at checkups. This is considered unnecessary exposure and should be avoided [46].

Dental imaging are low-radiation exposures except for the CBCT. Our study showed that few dentists were aware of the doses for different modalities, which could lead to confusion when communicating radiation risks to the patient [47]. Dentists may also fail to provide added protection when needed.

Protecting patients with lead aprons and thyroid collars has been a long-upheld safety measure. In the United States, a survey reported that 60% of dentists always use thyroid collars, and 76% use lead aprons [48]. In contrast, findings from the UK showed that lead aprons were not routinely used for dental imaging and that thyroid collars were used only with upper occlusal and CBCT images [10]. Similarly, a study in India reported that 90.3% of the participating dentists do not provide any protective wear for their patients [40]. A more recent study found that 63% of Indian participants were regularly using lead aprons and 82% were also using thyroid collars for patients during exposure [49]. A recent survey evaluating pediatric dentists in Europe reported that the thyroid collar was the most common radiation protection practice for patients during intraoral (75%) and panoramic radiographs (60%) [50]. While in our study, only 14.6% of the pediatric dentists reported using it for their patients. The wide variation in practice across countries, signals that recommendations are not clear or may not be adhered to.

Recently, several international bodies including the ADA, American Academy of Oral and Maxillofacial radiology and AADP no longer recommend the use of thyroid collars and lead aprons, for patients of all ages and conditions including pregnant women, provided that new-technology X-ray machines are available, that machines are well maintained and calibrated, and that correct patient position, dose limitation, procedure justification and optimization are observed. The rationale behind this recommendation is based on; new machines produce negligible radiation as well as PPE don’t prevent internal scattering and may interfere with imaging which requires retake. The use of adequate size rectangular collimators, digital films, and adhering to the ALARA principal, in addition to the above considerations, should be adequate for dose reduction. Meanwhile, the IAEA still recommends lead aprons for vertex occlusal imaging especially in pregnant women [51]. and the WHO has not updated the recommendation on the use of aprons. The generalizability of not routinely using protective wear during imaging will depend on the technology of the available X-ray machines and the patient’s preference.

In our study, Egyptian dentists prefer that the dentist or the patient support the film during intra-oral radiography compared to the use of film holders. Similarly, Aravind et al. showed that 71% of the dentists make their patients hold the film followed by 16.7% who use the film holders [40]. These finding differ from the results reported by Sheikh et al. where only 4.3% of dentists held the film themselves [26]. Another British study reported that 79.5% of specialists in comparison to 65.7% of GPs use film holders [45]. The use of film and sensor holders may improve the accuracy of imaging and reduce retakes [52], especially when small rectangular collimators are used.

When considering children, the “Image Gently” alliance has recommended the child-sized imaging protocols, due to their smaller body size, higher radiosensitivity and ongoing development [8]. The AAPD also states that age is not the defining factor of the timing of initial radiographs [14]. Yet twenty percent of the dentists in this study, thought that age was the primary determinant for requesting the initial radiographs. The unsupported practice of screening asymptomatic children in the mixed dentition with OPG [53] was followed by most survey participants.

Special consideration has always been given to pregnant women and to the protection of the fetus. Our results show that only 5.3% of respondents had knowledge of the specific 28-day rule applied to safe imaging of pregnant women. The recent recommendations, state that cancer risk to the unborn child and induction of genetic abnormalities are almost negligible compared to the background risk of cancer or genetic disorders [51].

A meta-analysis [43] reported large variations of effective doses for different CBCT units and field of view (FOV). The effective doses were between 84 mSv and 212 for adults depending on the FOV. While for children, the range was between 103 mSv and 175 mSv, in small and medium-large FOV, respectively. Another study evaluating absorption doses showed that the skin absorbed between 130 and 2818 mGy with panoramic radiography and between 328 and 11,994 mGy with CBCT imaging, for a single exposure [33]. The absorbed dose will account for much larger effective doses than those reported in the literature. Therefore, CBCTs should only be prescribed on individual basis with the smallest FOV, if conventional radiographs are inadequate to make decisions and if potential benefits outweigh the risk, especially in children [14, 17]. Similar to the knowledge reported in a survey done in Hong Kong [43], our findings showed low knowledge of the indications for CBCT imaging. Our survey showed a significant association between the presence of CBCT machines in facilities and dentists’ higher knowledge scores, which may suggest that advanced technology drives knowledge acquisition.

Several studies report that training dental students and professionals effectively improved radiation protection knowledge. Few previous surveys reported on the training received by dentists. Reports showed that 83% of Korean dentists [29] and 25.3% of Indian dentists [19] had completed a radiation safety program. In our survey, only 16.6% of Egyptian dentists had previous training. This low number of dentists may indicate the need for organizational adoption of operators’ continuing education (CE).

Most surveys reported participants’ enthusiasm towards training. A survey of Indian dentists showed that 94.3% were eager to enhance their knowledge. There was a direct association of the dentist’s attitude towards radiation safety with the level of education and practice type [19]. While a survey of Turkish dentists [18] reported no correlation between the attitudes of dentists and their age, speciality, or years of experience. Like these surveys, our results showed that dentists were willing to participate in CE programs. The results of this survey were considered with emphasis on the practice of pediatric dentists as their patients are the most sensitive to radiation and maxillofacial radiologists as they perform the most imaging.

### Limitations

At the time of the survey the number of practicing Egyptian dentists was 70,000 of which ≈50,000 practice in the Greater Cairo region. We distributed the survey to dental professionals’ platforms and Whats App groups. The survey reached approximately 4500 Egyptian dentists. However, 228 were eligible out of the 246 dentists who responded (≈5.5% response rate). This small response rate may indicate the disinterest in the topic by many dentists and further highlights the need to raise awareness on the importance of radiation safety. It may also signal that the questionnaire was too difficult to complete causing dentists to opt-out of submitting the survey [54].

Our sampling method was subject to selection bias. The self-selection of participants and the non-response bias from not having a full understanding of possible systematic characteristics of the non-responders as well as the coverage bias due to the inability to ascertain the portion of the population that was not reached may affect the representation of the total population. Such biases are inherent to the unsystematic convenience and snowballing sampling in online surveys [55, 56].

Many of the WhatsApp groups to which the survey was shared represented staff from universities and research organizations. This may be the reason for the greater number of academicians and researchers among the responders. However, the professional dental platforms with approximately 4500 dentists were mostly dentists in private practice and governmental hospitals. Fewer dentists responded from these groups.

In the future, more accurate generalizability of online surveys can be achieved by adopting random systematic sampling. This may be achieved by random sampling from a closed population as dental schools or various dental associations that have a registry or membership list. This facilitates the creation of a stratified sampling frame to include different subgroups, accurately calculate the response rate and evaluate the reasons for non-responses [55, 56]. This, however, is not always possible due to privacy issues.

The aim of KAP surveys is to generate knowledge to inform better practice. A closer look at the published surveys showed variability of questions, as well as lack of validation and information on the recommended Diagnostic Reference Levels. This can suggest the need for the development and testing of a standard survey tool that measures knowledge in a way that may better interpret and impact radiation safety practices.

## Conclusion

Within the limitations of this study, the following conclusions can be made from the results of our survey in light of recent guidelines:

1. There is a need to educate dentists on radiation hazards, child-sized imaging protocols and how to implement the ALARA principle.
2. There is a need to update school radiology curricula, for institutes to provide continuing education programs and to disseminate the free training materials available on reliable websites.
3. A radiation safety culture should be adopted, and periodic monitoring and auditing should be included in facility radiation safety programs.

## Supplementary information

STROBE checklist

## Data Availability

The data that support the findings of this study are not openly available but can be provided by the corresponding author upon reasonable request.
